# Neutrophil PAD4: how does it function in cancer beyond promoting NETosis?

**DOI:** 10.18632/oncotarget.28369

**Published:** 2023-03-24

**Authors:** Laura Garcia-Gerique, Yulia Nefedova

**Keywords:** PAD4, PMN-MDSC, neutrophils, neutrophil migration

Expansion of pathologically activated immune suppressive myeloid cells called myeloid-derived suppressor cells (MDSC) is one of the hallmarks of cancer. In most tumor types, MDSC are represented primarily by pathologically activated neutrophils (PMN-MDSC) [1]. PMN-MDSC originate in the bone marrow and migrate to various sites including tumor tissues and premetastatic niches. These cells have a relatively short lifespan (less than 48 hours) and, therefore, are continually replaced from the bone marrow [2]. Tumor-infiltrating PMN-MDSC possess a potent suppressive activity as they are able to inhibit both antigen-specific immune responses of T cells and non-specific anti-CD3/CD28-stimulated responses [3]. As a result, a highly immunosuppressive environment is created in tumors, which prevents their rejection via immunological mechanisms. In addition, PMN-MDSC employ non-immunological mechanisms to facilitate tumor progression, including angiogenesis, remodeling of extracellular matrix, and production of cytokines. In patients with solid tumors, levels of MDSC in circulation and tumor tissues have been positively associated with a poor response to the therapy in many types of cancer and represent an independent indicator of poor outcomes [4]. However, many of the details about how PMN-MDSC support cancer progression, and thus approaches for therapeutically targeting these cells, remain enigmatic.

Neutrophils can form neutrophil extracellular traps (NETs), an extracellular web-like structure consisting of fibers of chromatin and proteins normally found inside neutrophil granules, nucleus, or cytoplasm. NETosis, a process of NET formation, was initially described in 2004 by Brinkmann and Zychlinsky as a mechanism by which activated neutrophils can kill bacteria [5]. Since then, a role of NETs has been demonstrated for a number of pathological conditions including cancer. Recent studies suggest that NETs may have a pro-tumoral effect, mediated by their ability to promote cancer cell proliferation, awake tumor dormant cells, or sustain immune evasion. In cancer patients, the presence of NETs associates with advanced cancer stage, poor prognosis, and metastatic disease [6]. Multiple studies including ours have previously reported that peptidylarginine deiminase 4 (PAD4) is a major factor regulating NETosis in cancer [7]. PAD4, an enzyme responsible for citrullination of multiple target proteins, is highly expressed by neutrophils. PAD4 has been implicated in NETosis [8] due to its ability to citrullinate arginine residues of histone H3, ultimately contributing to chromatin decondensation, one of the pre-requisites for NET formation. However, only a fraction of neutrophils is undergoing NETosis [9] and our knowledge about the function of neutrophil PAD4 in cancer beyond its involvement in NET formation remains limited. In our recent study [10], we identified a novel mechanism by which neutrophil PAD4 promotes cancer progression.

Using several transplantable and genetically engineered mouse models, we demonstrated that tumor growth was accompanied by significantly elevated enzymatic activity of neutrophil PAD4 [10]. To further clarify the role of PAD4 in tumor progression, we utilized PAD4^fl/fl^ MRP8^Cre^ mice with targeted deletion of PAD4 in myeloid cells, primarily neutrophils. Lack of neutrophil PAD4 delayed the growth of primary tumors and dramatically reduced lung metastases. At the same time, no decrease in immune suppressive activity of tumor-infiltrating and spleen PAD4-deficient neutrophils was observed defining these cells as PMN-MDSC. However, targeted deletion of PAD4 in neutrophils did markedly decrease the intratumoral abundance of (PAD4-deficient) neutrophils; this decrease was not due to a decline in bone marrow or spleen myelopoiesis or to neutrophil death due to NETosis. Given this substantial reduction in the number of neutrophils in primary tumors and lungs upon PAD4 depletion, we hypothesized that the lack of PAD4 in neutrophils may reduce their motility and, therefore, impair their recruitment to the tumor site and pre-metastatic niche. Indeed, targeted deletion or pharmacological inhibition of PAD4 significantly reduced migration of neutrophils in response to the chemokine CXCL1 without suppressing spontaneous migration or migration induced by the chemoattractant fMLP.

To explore how PAD4 supported neutrophil migration, we asked whether PAD4 activity correlated with expression of the two major cell surface receptors responsible for the regulation of neutrophil trafficking between the bone marrow and tissues, CXC motif chemokine receptor 2 (CXCR2) and CXCR4. Whereas CXCR4 promotes neutrophil retention in the bone marrow, upregulation of CXCR2 leads to release of neutrophils into the circulation and migration to the tissues [11]. We found that PAD4 promoted neutrophil accumulation in tumor and metastatic tissues by regulating the expression of the chemokine receptor CXCR2. CXCR2 surface levels are known to be modulated by two mechanisms: cell internalization and recycling upon ligand binding, and by a metalloprotease activity of ADAM17 upon neutrophil activation by nonligand stimuli. However, little has been known about factors that regulate CXCR2 transcription. The PAD4 enzyme is localized to the cytoplasm and nucleus and citrullinates multiple target proteins including histones, as noted above, as well as transcriptional factors and co-factors. Our data indicated that PAD4 regulates CXCR2 expression on transcriptional level. Although, the precise mechanism responsible for this remains to be defined, our work demonstrates that PAD4 in neutrophils supports their migration to tumors by upregulating a key cytokine receptor involved in trafficking, CXCR2.

**Figure 1:**
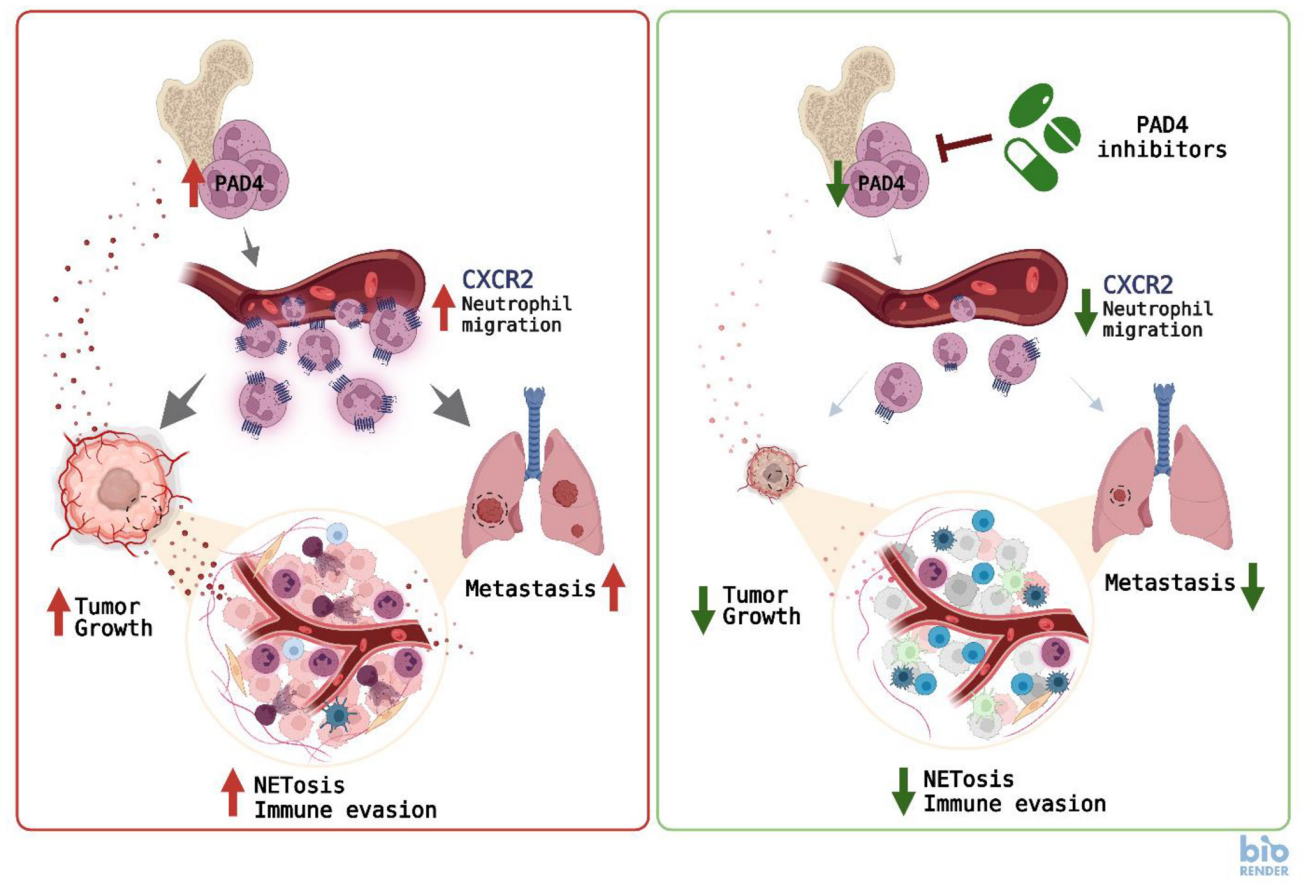
Mechanisms involved in regulation of tumor progression by neutrophil PAD4. Left: Tumor-derived factors induce activation of the PAD4 enzyme in bone marrow neutrophils leading to upregulation of CXCR2 expression, release of neutrophils into the circulation, and their migration to the primary tumor tissues and metastatic sites. A high gradient of tumor-derived factors in the tumor microenveronment stimulates neutrophils to produce NETs. The abundance of immune suppressive neutrophils and NETs in the tumor microenvironment suppress antitumor immune responses, thus, promoting tumor growth and metastases. Right: Inhibition of neutrophil PAD4 downregulates CXCR2 expression leading to decreased neutrophil migration, reduced presence of neutrophils in the tumor tissues, and activation of immune responses. This ultimately results in delayed growth of primary tumors and dramatically reduced metastastatic disease.

We demonstrated that targeted deletion or pharmacological inhibition of PAD4 enhanced the anti-tumor effect of checkpoint inhibitors in mouse models of metastatic cancers. Pre-clinical studies showed a benefit of blocking neutrophil recruitment to the tumor tissues by CXCR2 inhibition, particularly in combination with immunotherapy [12]. Several CXCR1/2 inhibitors, including reparixin, Navarixin, or SX-682 have been developed and tested in early phase clinical trials in combination with immune checkpoint inhibitors. However, clinical results have not been impressive so far. Signaling pathways involved in cancer immune evasion are redundant and blocking one signaling pathway may induce compensatory mechanisms. Thus, rational design of drug combinations targeting complementary tumor-promoting mechanisms will likely produce the most effective therapeutic approach. In this respect, blocking PAD4 has a clear advantage over selectively blocking CXCR1/2 as PAD4 blockade also leads to the inhibition of NETosis and potentially of other receptors/signaling pathways that have yet to be identified (Figure 1).

Taken together, our study identified a new mechanism responsible for transcriptional regulation of neutrophil migration and a new mechanism by which neutrophil PAD4 is contributing to tumor progression. PAD4 inhibitors are in development and may enter early phase clinical trials in the future. This will open a novel therapeutic approach for targeting PMN-MDSC in cancer.
